# Top-Down Effect of Arthropod Predator Chinese Mitten Crab on Freshwater Nutrient Cycling

**DOI:** 10.3390/ani13142342

**Published:** 2023-07-18

**Authors:** Lin Wang, Hongjun Liu, Francisco Carvalho, Yunru Chen, Linshiyu Lai, Jiachun Ge, Xingjun Tian, Yunchao Luo

**Affiliations:** 1 School of Life Sciences, Nanjing University, 163 Xianlin Avenue, Nanjing 210023, China; 2 CBMA-Center of Molecular and Environmental Biology, Biology Department, University of Minho, 4710-057 Braga, Portugal; 3 Beijing Municipal Ecological and Environmental Monitoring Center, 14 Chegongzhuangxi Road, Beijing 100048, China; 4 Freshwater Fishery Institute of Jiangsu Province, Nanjing 210017, China

**Keywords:** freshwater ecosystem, litter decomposition, ecoenzymatic stoichiometry, trait-mediated indirect interaction, invasive species

## Abstract

**Simple Summary:**

Crabs are advanced predators in aquatic ecosystems, and they can feed directly on plant litter. They can also indirectly regulate the decomposition of organic matter in the aquatic environment by influencing snail populations and the structure of the substrate microbial community through top-down effects. When the invasive organism channeled apple snail (*Pomacea canaliculata*) meets the native species Chinese mitten crab (*Eriocheir sinensis*), new interspecific relationships and food web structures are created. This is of great concern in the context of climate change.

**Abstract:**

Aquatic litter decomposition is highly dependent on contributions and interactions at different trophic levels. The invasion of alien aquatic organisms like the channeled apple snail (*Pomacea canaliculata*) might lead to changes in the decomposition process through new species interactions in the invaded wetland. However, it is not clear how aquatic macroinvertebrate predators like the Chinese mitten crab (*Eriocheir sinensis*) will affect the nutrient cycle in freshwater ecosystems in the face of new benthic invasion. We used the litter bag method to explore the top-down effect of crabs on the freshwater nutrient cycle with the help of soil zymography (a technology previously used in terrestrial ecosystems). The results showed significant feeding effects of crabs and snails on lotus leaf litter and cotton strips. Crabs significantly inhibited the intake of lotus litter and cotton strips and the ability to transform the environment of snails by predation. Crabs promoted the decomposition of various litter substrates by affecting the microbial community structure in the sediment. These results suggest that arthropod predators increase the complexity of detrital food webs through direct and indirect interactions, and consequently have an important impact on the material cycle and stability of freshwater ecosystems. This top-down effect makes macrobenthos play a key role in the biological control and engineering construction of freshwater ecosystems.

## 1. Introduction

Freshwater ecosystems emit and bury substantial amounts of carbon [1], making a significant contribution to the carbon cycle on a global scale [2]. The release of carbon dioxide in freshwater ecosystems mainly comes from the decomposition of allochthonous organic matter by microorganisms and the respiration of animals and plants [3]. Furthermore, the input and decomposition processing of plant litter can cause changes in the water environment, sediment nutrient content, enzyme stoichiometry, and other ecological indicators [4,5]. The continuous availability of water and nutrients in aquatic ecosystems enhances metabolic rates and promotes the decomposition process of organic matter in water [6]. Therefore, as a key process of the material cycle and energy flow [7,8], aquatic litter decomposition is a tool to evaluate the functional integrity of freshwater ecosystems [9,10].

Litter decomposition in freshwater ecosystems is affected by the climate and environment [11], litter quality and diversity [12], or the biota [13]. Aquatic organisms, especially macroinvertebrates (including crustaceans) and microorganisms, play a major role in nutrient cycling in freshwater ecosystems by directly feeding on plant litter from riparian plant vegetation or aquatic macrophytes [14]. When litter first enters the water channel, it is colonized by microbial communities that produce extracellular enzymes, which promote the catalysis of recalcitrant substances and improve the palatability of litter to invertebrates [15]. The next stage of decomposition occurs when coarse organic matter is processed into fine particulate organic matter, which has a pivotal importance for other aquatic organisms through the food chain, including nutrient supply for plants [16,17]. Therefore, the richness and abundance of benthos will directly or indirectly affect nutrient cycling in freshwater ecosystems [18]. Additionally, the feeding, movement, and defecation of benthic macroinvertebrates disturb the sediment, which may also affect microbial community structure in the water and sediment. [19].

Many studies have found that alien invasive organisms have changed the interactions between species in the original environment [20], which may ultimately affect ecosystem processes, such as litter decomposition [21]. The channeled apple snail (*Pomacea canaliculata*), native to the tropical and subtropical regions of South America, has become a worldwide invasive species, including China. Without natural enemies, channeled apple snails have caused economic harm by impacting agricultural activities, such as rice production. Parallelly, channeled apple snails also have caused important ecological impacts on freshwater ecosystems and their processes, such as litter decomposition, but those have not received as much attention. As an omnivore, the native Chinese mitten crab (*Eriocheir sinensis*) presents large feeding plasticity [22]. With a strong claw closing force and strong aggression [23], they can act as successful benthic predators for mollusks (such as snails and mussels with hard shells). But they are also able to feed on plant material, including macrophytes or leaf detritus. Currently, with the intensification of climate changes (e.g., global warming), the suitable living areas of various organisms, including channeled apple snails [24], have further expanded to low latitudes [25,26]. Those geographic changes have been consequently increasing the sympatric distribution between the invasive channeled apple snails and the native Chinese mitten crab, enhancing the importance of studying interactions between both species and the resulting impacts on important ecosystem functions and nutrient cycles in freshwater ecosystems.

While the feeding behavior of benthic animals is important for the fragmentation of organic matter, such as aquatic plant litter, microorganisms are the ultimate performers of carbon transformation. Persson et al. [27] estimate that at least 95% of the energy flow needs to pass through microbial populations. Studies on nutrient cycling in freshwater ecosystems have proven that extracellular enzymes secreted by microorganisms play an important role in the decomposition of organic matter. For example, sediments from peat fens exhibit high cellulase activity, and these are mainly secreted by microorganisms and meiobenthos (Oligochaeta species) [28]. Given the close correlation between extracellular enzymes and nutrients, the activity of some enzymes is often used as an indicator of nutrient deficiency. β-1,4-glucosidase (BG), β-1,4-*N*-acetylglucosaminidase (NAG), and acid phosphatase (AP) can serve as indicators, respectively, of C, N, and P nutrient deficiency and microbial nutrient demand [29,30]. Therefore, the investigation of microbial population changes and community structure characteristics, as well as substrate ecoenzyme stoichiometry, is essential for a better understanding of aquatic ecosystem biological processes and carbon cycling. However, most of the traditional research methods for studying enzyme activity directly sample the water or sediment sampling points, and such an approach makes it difficult to avoid the problem of environmental heterogeneity at sampling sites. We applied a method in terrestrial ecosystems, soil zymography, to the detection of ecological enzyme activity and sediment hotspots from a two-dimensional plane.

We aimed to interpret the ecological top-down effect of arthropod predators in the freshwater nutrient cycle through the two-dimensional spatial distribution characteristics of enzymes. We predict that: (1) Macrobenthic predators not only slow the decomposition of sediment organic matter through density-mediated indirect interactions, but also affect the nutrient cycle rate of freshwater ecosystems through trait-mediated indirect interactions. Considering diet selection of animals are usually stable, the channeled apple snail is an important food of its native (Central and South America) natural enemy, the snail kite (*Rostrhamus sociabilis*), but the Chinese mitten crab may not recognize the exotic channeled apple snail as potential prey. Thus, we also predict that: (2) the Chinese mitten crab has less influence on the alien invasive species channeled apple snail compared with the native species *Radix auricularia*.

## 2. Materials and Methods

### 2.1. Subjects and Feeding Conditions

In this study, Chinese mitten crabs were collected from the Jintan Cooperation Base of the Freshwater Fishery Institute in Changzhou City, Jiangsu Province, China, in March 2021. Only healthy, active, and intact crabs were selected. *R. auricularia* were collected from the Shenfengyuan aquaculture farm in Huai’an, Jiangsu, and *P. canaliculata* from the Channeled apple snail aquaculture farm in Yangjiang, Guangdong. All the animals were transported to the laboratory and kept in a large plastic water tank (80 × 60 × 23 cm) before the experiment. To ensure the health of the experimental animals, 12 crabs or 100 snails were kept in each water tank. Animals were fed (Crab: pellet feed; snails: naturally dried lotus leaf litter, collected in December 2020), and the water (temperature 20–25 °C) was changed once a day.

### 2.2. Crab Feeding Preference Experiment

To explore the feeding preference of crabs for two kinds of snail species, we conducted a predation selection test. The experiment was carried out with small plastic water tanks (34 × 22 × 10 cm) and stones included to be used as shelters. There were 20 replicates, and we added 1 crab to each water tank and allowed animals to adapt to the water tank for 1 week, adding pellets as food resources and changing the water (50%) once a day.

At the beginning of the experiment, 5 *R. auricularia* individuals and 5 *P. canaliculata* (the average weight of *R. auricularia* in each water tank ≈ that of *P. canaliculata*, Mann–Whitney U tests *p* > 0.05, Table S1) were added to each water tank, and 2 g of lotus leaf litter was added. To exclude the natural death of snails without crab predation, we added another 20 replicates as the control group. Except for crabs, which were not added, other operations were consistent with the experimental group. After that, the survival numbers of the two kinds of snails were counted, and the water was changed once a day. The experimental period was 15 days.

### 2.3. Litter Decomposition Experiment

#### 2.3.1. Experimental Design

We used the standing withered leaf litter of the common aquatic plant lotus (Nelumbo nucifera Gaertn.) as a natural decomposition substrate. Cotton strips (size: 8 × 3 cm) and flat wood sticks (wooden tongue depressors made from *Betula* sp.; size: 15 × 1.8 cm) with fixed and uniform components were selected as standard litter substitutes (hereafter collectively referred to as standard litter) [31]. The main component of cotton strips is cellulose, and the main components of wood sticks are lignin, cellulose, and hemicellulose. Standard litter reflects the ecological process of litter decomposition more accurately than natural decomposition substrates [32]. To distinguish the decomposition of standard litter by benthic fauna and microorganisms, we used litter bags with different mesh sizes. The mesh size of the coarse mesh litter bags was 8 mm (allowing experimental animals to enter or gnaw), and the mesh size of the fine mesh litter bags was 0.2 mm (to measure separately the contribution of microorganisms avoiding the entry of experimental animals). One cotton strip, one wood stick, and 1.5 g of lotus leaf litter were placed into each litter bag.

The experiment was carried out with six large plastic water tanks (80 × 60 × 23 cm). Eight kilograms of lotus pond mud (purchased from Tianfeng nutrient soil store, Huai’an City, Jiangsu Province) and 50 L of lotus pond water (from Lizhao Lake of Nanjing University Xianlin campus) were added to each water tank. All the components were allowed to acclimate to experimental conditions for 1 week before the experiment. We had six treatments: (A) only litter; (B) litter + *R. auricularia*; (C) litter + *P. canaliculata*; (D) litter + Chinese mitten crab; (E) litter + *R. auricularia* + Chinese mitten crab; and (F) litter + *P. canaliculata* + Chinese mitten crab. One large water tank was used for each treatment. The total number of litter bags was 60 (6-treatment × 2-mesh size × 5-litter bag repetition). At the beginning of the experiment, 100 *R. auricularia* individuals were added to groups B and E, 100 *P. canaliculata* individuals were added to groups C and F (the average weight of *R. auricularia* ≈ that of *P. canaliculata*, Table S2), and 5 Chinese mitten crab individuals were added to groups D, E and F. To maintain the same predation ability and food intake of crabs in different treatments, male crabs were selected for all groups, and the average weight of crabs in each group was roughly the same (38.08 ± 0.88 g). During the experiment, distilled water was added to the standard water level (50 L, water depth of about 10 cm) every day. Since the snails die naturally, from the third week, 20 *R. auricularia* were added to groups B and E every week, and 20 *P. canaliculata* were added to groups C and F (the average weight of *R. auricularia* ≈ that of *P. canaliculata*, Table S2).

#### 2.3.2. Sample Recovery and Testing

Water quality parameters, including pH, conductivity (COND), and total dissolved solids (TDS, an essential indicator of surface water quality) [33], were measured by a LICHEN PH-100 once a day. The substrate-induced respiration (SIR) of water was tested once a week. Five milliliters of water were taken by the five-point sampling method, and the CO_2_ content was measured by a Korno gas detector (GT-903). After 6 weeks of the decomposition experiment, the litter bags were recovered, washed, dried at 60 °C, and weighed, and the litter mass loss was calculated:

$$Mass\;loss\left(\%\right)=\left(Final\;mass-Initial\;mass\right)/Initial\;mass\times 100\%.$$

Before recovering litter samples, we collected the surface sediment for macrogenomic sequencing (Supplementary Materials). One mixed sample for each treatment group was collected by using a syringe to suck 1 mL × 5-sediment on a five-point sampling method, and the sample was mixed evenly. Samples were submitted to Sangon Company (Shanghai, China) for paired-end metagenomic sequencing on an Illumina HiSeq sequencing platform.

We drained the water in the water tank, dried it naturally, and monitored the humidity with a soil conductivity sensor (Takeme) until the humidity was approximately 20%. The soil conductivity was detected by the soil conductivity sensor. We used soil zymography [34,35] to test the enzyme activity and hotspots of acid phosphatase (AP), β-1,4-glucosidase (BG), and β-1,4-*N*-acetylglucosaminidase (NAG). One gram of soil was taken for the SIR test. The sediment was thoroughly air-dried, and 10 g samples were taken to detect soil organic carbon (SOC), total nitrogen (TN), and total phosphorus (TP; Supplementary Materials). A pH Meter PHS-3C was used to determine the sediment pH according to the method of HJ962-2018 [36]. The above steps were based on the five-point sampling method, *n* = 5.

### 2.4. Data Analysis

The Wilcoxon signed-rank test was used to verify the selectivity difference of crabs to two kinds of snails in the crab feeding preference experiment, and a paired t-test was used to verify the selectivity in the early stage of the experiment (1–6 days).

MATLAB R2020a was used to analyze the average enzyme activity and hotspot area of soil zymography (Supplementary Material; Figure S1). We calculated the vector of enzyme stoichiometry [30]. The calculation formulas of vector length (L) and vector angle (A) were as follows [37]:

$$L={\left[{\left(lnBG/lnNAG\right)}^{2}+{\left(lnBG/lnAP\right)}^{2}\right]}^{1/2};$$

$$A=Degrees\left[ATAN2\left(lnBG/lnAP,lnBG/lnNAG\right)\right].$$

The longer L is, the greater the carbon limit; A < 45° and >45° represent the relative degree of nitrogen limitation or phosphorus limitation, respectively. The greater the deviation is, the stronger the limitation [38]. Enzyme C/N was characterized by lnBG/lnNAG, enzyme C/P by lnBG/lnAP, and enzyme N/P by lnNAG/lnAP. All the results are the mean ± SE.

The effects of the treatment, litter material, and mesh size of the litter bags on mass loss were analyzed by a generalized linear model (GLM). The three factors were used as fixed factors. To meet the normal distribution of data assumption, the mass loss was ln (X + 1) transformed. The LSD test was used to analyze the effects of the treatment on the decomposition rate of different litter, water quality, and sediment parameters. Pearson correlation analysis was carried out between the mass loss in fine mesh litter bags and various sediment indexes. We used R language (vegan package) to perform a Non-metric multidimensional scaling analysis (NMDS), analyzing the differences in microbial taxonomy and carbohydrate-active enzymes of different treatments. In addition, we also used R language (hier.part package) to perform hierarchical partitioning [39], analyzing the contribution of nutrients and their related enzymes to the litter decomposition rate in fine mesh litter bags.

All statistics were performed using R4.1.0, and two-tailed probabilities of 0.05 were considered significant. The Graphical Abstract was drawn using Adobe Illustrator 2021 (25.0.1 for Mac), and other figures were drawn using OriginPro 2022 SR1 (9.9.0.225).

## 3. Results

### 3.1. Predation of Chinese Mitten Crab on Both Snail Species

**Figure 1:**
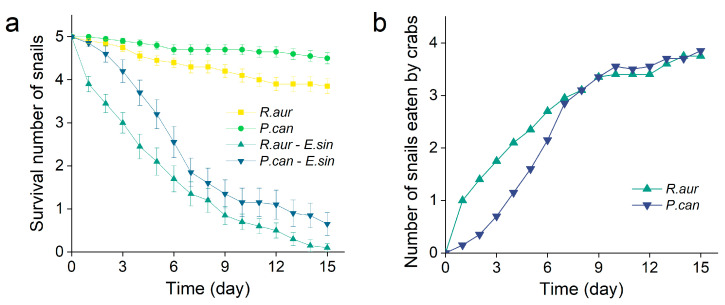
Selective predation of crabs on snails. (**a**,**b**) The results of the feeding preference of the Chinese mitten crab on *R. auricularia* and *P. canaliculata*. Figure (**a**) is the survival curve of snails under four treatments, and (**b**) is the number of prey on snails by crabs. The number of replicates is *n* = 20.

In the early stage of the crab feeding preference experiment (1–6 days), the Chinese mitten crabs preferentially predated *R. auricularia* (t = 10.938, df = 5, *p* < 0.001), which was 85.2% higher than that of *P. canaliculata* (Figure 1). Predation of crabs on *P. canaliculata* increased with the decrease in *R. auricularia* (after 7 days). Finally, there was no quantitative difference in the selective predation of crabs between both snail species (z = −1.543, *p* = 0.123). This means that the invasive species *P. canaliculata* is not the preferred food of the Chinese mitten crab.

### 3.2. Dynamic Changes in the Water Quality Parameters of the Decomposition System

**Figure 2:**
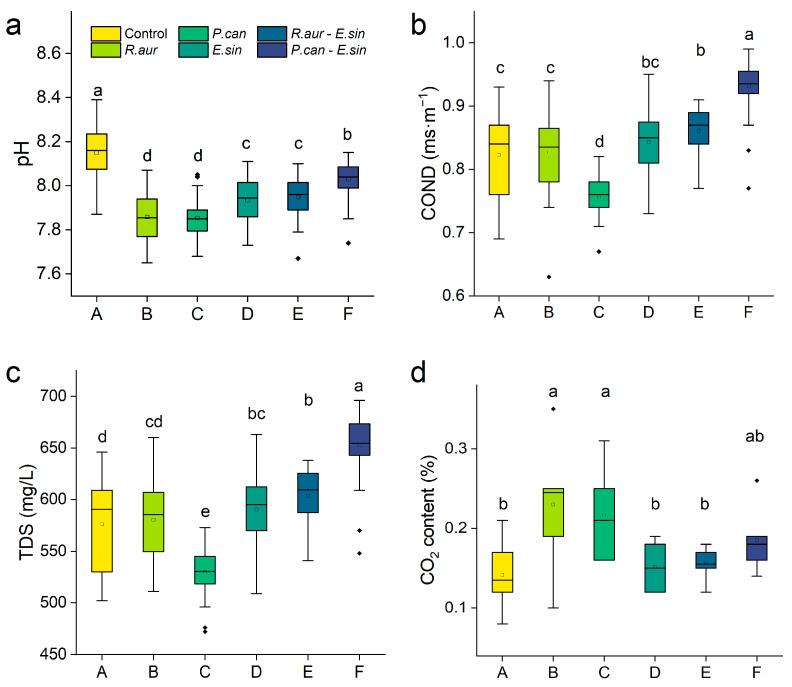
Water quality parameters in the litter decomposition experiment. (**a**–**d**) The water quality parameters such as pH, conductivity (COND), total dissolved solids (TDS), and substrate-induced respiration (SIR) in the decomposition system. The capital letters A–F on the abscissa axis represent the six treatments. Different lowercase letters in the same figure represent significant differences in LSD tests. The sample size is *n* = 44 or *n* (SIR) = 6. *R. aur* in the legend is the native species *R. auricularia*, *P. can* is the invasive species *P. canaliculata*, and *E. sin* is the predator *E. sinensis*.

The participation of experimental animals obviously affected the water quality parameters in the decomposition system (Figure 2). The LSD test showed that both snail species significantly reduced the pH of the water and improved the SIR (*p* < 0.05; Figure 2a,d; Table S3). In addition, *P. canaliculata* also significantly reduced the conductivity and TDS (*p* < 0.05, Figure 2b,c). In contrast, the addition of crabs significantly improved the conductivity and TDS of water (*p* < 0.05; Figure 2b,c).

### 3.3. Decomposition Rate of Lotus Leaf and Standard Litter

**Figure 3:**
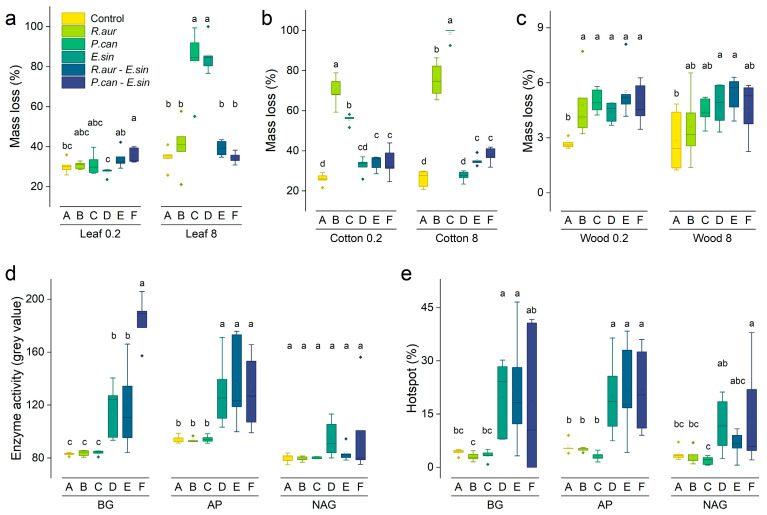
Litter decomposition rate and soil zymography analysis. (**a**–**c**) The decomposition rates of lotus leaf litter, cotton strips, and wood sticks after 6 weeks of decomposition. Numbers 0.2 and 8 in the abscissa axis represent the mesh size of the litter bag. (**d**) Soil enzyme activity of sediment after 6 weeks of decomposition. (**e**) The corresponding enzyme hotspot area. BG in the abscissa axis is β-1,4-glucosidase, AP is acid phosphatase, and NAG is β-1,4-*N*-acetylglucosaminidase (the same below). The capital letters A–F on the abscissa axis represent the six treatments. Different lowercase letters in the same treatment represent significant differences in LSD tests.

The results of the GLM showed that when the three benthos existed alone (Group B, C, and D), they significantly accelerated the rate of litter decomposition (*p* < 0.05; Figure 3; Table S4). The direct feeding of *P. canaliculata* (Group C) and crabs (Group D) increased the mass loss of lotus litter in coarse mesh litter bags by 140.2% and 147.8%, respectively (Table S5). The direct feeding of *R. auricularia* (Group B) and *P. canaliculata* (Group C) increased the mass loss of cotton strips in coarse mesh litter bags by 190.1% and 278.7%, respectively. The microbial decomposition process in fine mesh litter bags was also significantly affected by the two kinds of snails (*p* < 0.05; Table S5). The decomposition rate of cotton strips in fine mesh litter bags increased by 169.1% and 115.1%, respectively, in *R. auricularia* (group B) and *P. canaliculata* (Group C) individual treatments. In addition, the decomposition rate of wood sticks was significantly lower than that of lotus litter and cotton strips (t = −18.467, *p* < 0.001). The decomposition rate in fine mesh litter bags was significantly lower than that in coarse mesh litter bags as a whole (t = −4.221, *p* < 0.001), but the mass loss of wood sticks in fine mesh litter bags in the treatments with snails (Groups B, C, E and F) was higher than that in coarse mesh litter bags (Table S5). Overall, benthic invertebrate interactions influence the rate of litter decomposition.

### 3.4. Correlation between Sediment Properties and Litter Decomposition

**Figure 4:**
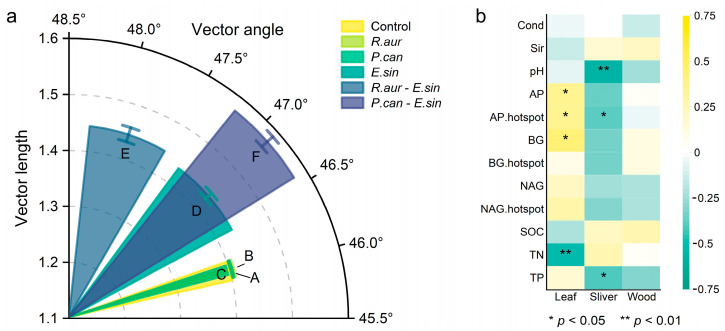
Enzyme stoichiometry of sediment and correlation analysis between sediment properties and litter decomposition. (**a**) The result of enzyme stoichiometry of sediment. The longer the vector length is, the greater the carbon limit; vector angles < 45° and >45° represent the relative degree of nitrogen limitation or phosphorus limitation, respectively. Since all of the vector angles are >45°, it means that the sediment primarily has phosphorus limitation. The greater the deviation is, the stronger the limitation. The value is the mean ± 1/2 SE, and the sample size is *n* = 5. Figure The capital letters A–F on the abscissa axis represent the six treatments. (**b**) The correlation analysis between the soil parameters of the lotus pond mud and the decomposition rate in the fine mesh litter bags. Color depth represents the size of the correlation coefficient. The darker the color is, the greater the correlation coefficient.

The results of soil zymography showed that neither snail species affected enzyme activity in the sediment or hotspot areas when they were the only consumer present (Figure 3 and Table S6). The participation of crabs significantly increased the enzyme activities and hotspots of acid phosphatase (AP), and β-1,4-glucosidase (BG), but the effect on β-1,4-*N*-acetylglucosaminidase (NAG) was relatively small (Table S6). At the same time, the enzyme stoichiometric vector changed: when *R. auricularia* and crab coexisted (Group E), the sediment phosphorus (P) limitation increased significantly; when the *P. canaliculata* and crab coexisted (Group F), the sediment C limitation increased significantly (Figure 4a, Table S6). In addition, correlation analysis showed that the mass loss of lotus litter in fine mesh litter bags was significantly positively correlated with AP enzyme activity, AP hotspot area, and BG enzyme activity, while the mass loss of cotton strips was significantly negatively correlated with AP hotspot area (*p* < 0.05; Figure 4b).

After 6 weeks of decomposition, the physical and chemical properties of the sediment changed; the SIR of sediment increased significantly with the coexistence of *R. auricularia* and crabs (Group E), while the total nitrogen (TN) of sediment decreased significantly with the coexistence of *P. canaliculata* and crabs (*p* < 0.05; Group F). In addition, the sediment pH of groups B, C, and F decreased significantly (*p* < 0.05; Table S6).

### 3.5. Changes in the Microbial Community

**Figure 5:**
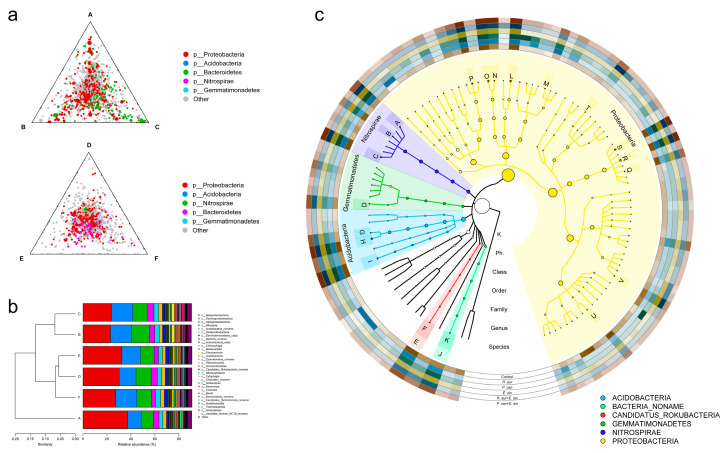
Macrogenome sequencing of sediment after litter decomposition. (**a**) The ternary phase diagram at the genus level. There were more specific genera in groups A, B, and C without crabs, while groups D, E, and F with crabs were more aggregated and unified. Figure (**b**) is the clustering diagram at the class level. Groups B and C without crabs were farther away from control group A. Figure (**c**) shows the hierarchical annotation of GraPhlAn at the species level. There were more unique microbial species in groups B and C without crabs (A: Nitrospira_defluvii, B: Nitrospira_sp_SCGC_AG-212-E16, C: Nitrospira_moscoviensis, D: Gemmatirosa_kalamazoonesis, E: Candidatus_Rokubacteria, F: Candidatus_Rokubacteria_bacterium_CSP1-6, G: Acidobacteria_bacterium_OLB17, H: Acidobacteria_bacterium_DSM_100886, I: Pyrinomonas_methylaliphatogenes, J: Bacteria_noname, K: uncultured_bacterium, L: Steroidobacter_denitrificans, M: Lysobacter_dokdonensis, N: Gammaproteobacteria_bacterium_SG8_31, O: Gammaproteobacteria_bacterium_SG8_30, P: Sphingomonas_jaspsi, Q: Betaproteobacteria_bacterium_SG8_41, R: Betaproteobacteria_bacterium_SCGC_AG-212-J23, S: Betaproteobacteria_bacterium_SG8_39, T: Sulfuritalea_hydrogenivorans, U: Ramlibacter_tataouinensis, and V: Methylibium_sp_Root1272).

The results of metagenome sequencing showed that the experimental animals affected the microbial community of the sediment (Figure 5b). The treatments were clustered according to the relative abundance of the microbiota level. The difference between group BC with snails only and control group A was the largest. The microbial communities in treatment groups A, B, and C without crabs had stronger specificity at the genus level, while the ternary phase diagrams of other treatment groups with crabs were more aggregated (Figure 5a). GraPhlAn showed that the BC group without crabs had more unique microbial species, especially some groups in Proteobacteria and Bacteroidetes. The NMDS analysis showed relatively more consistent and concentrated sample points in the treatments where crabs were involved (treatments D, E, and F) for both taxonomy and carbohydrate-active enzymes (Figure S2).

## 4. Discussion

Our results clearly demonstrate the top-down effect of macrobenthic predators in the food web. Chinese mitten crab not only feeds directly on litter in sediment but also affects the decomposition rate of organic matter by acting on small animals and microorganisms. The results highlight that the native macroinvertebrates are crucial in controlling alien invasion, although the exotic channeled apple snail is more stubborn than the native species *R. auricularia*.

Our results were consistent with the study of Turke et al. [40], which suggests that snails play a decisive role in litter decomposition in freshwater ecosystems. Especially for high-quality substrate, there was a strong direct feeding effect of both snail species on cotton strips and lotus litter. This effect not only accelerated the nutrient cycle but also increased the release of greenhouse gases. It is worth mentioning that in the treatment with snails, the decomposition of wood sticks in the fine mesh litter bag was faster than those in the coarse mesh litter bag. Wood sticks have poor palatability for animals, and their mass loss mainly comes from the catalytic degradation of microbial extracellular enzymes. As scraping eaters, snails easily eat microorganisms on wood sticks, resulting in less mass loss of wood sticks in coarse mesh litter bags.

As the top layer of the benthic food chain, Chinese mitten crabs can not only cause litter mass loss directly through feeding but also indirectly affect litter decomposition through the food chain. We suggest that the top-down indirect effects cannot be ignored. This top-down effect was divided into the density-mediated indirect interaction [41] and the trait-mediated indirect interaction [42,43]. First, the predation of snails by crabs led to a reduction in the consumption of litter by snails (Figure 3), which was similar to the study of Ewers et al. [44]. Second, the metabolites and behavioral activities of crabs changed the quantity and distribution of resources in the aquatic environment. Chen et al. [45] found that the presence of crabs is positively correlated with soil C/P and N/P ratios, which is inconsistent with our results (Table S6). In our experiment, changes in the environment made the microbial community structure more aggregated (Figure 5). The relative abundance of some taxa in Proteobacteria and Bacteroidetes was greater in the absence of crabs, while Betaproteobacteria were significantly inhibited by two species of field snails. Changes in microbial community structure also affected the secretion of microbial extracellular enzymes and indirectly affected litter decomposition. Trait-mediated indirect interactions are usually more difficult to find and judge than density-mediated indirect interactions. For example, Hawlena et al. [46] found that the presence of arthropod predators changes the C:N of prey and further affects subsequent litter decomposition. The ecological top-down effect caused by this potential indirect effect might be more prominent when alien organisms invade [47].

The lack of natural enemies makes it easy for invasive organisms to change the biological community structure and ecological process in the invaded area [48]. Our results showed that Chinese mitten crabs preferred to eat native species, i.e., *R. auricularia* (Figure 1). However, the decomposition ability of channeled apple snails to various substrates is great, including its direct feeding and the indirect effects of environmental and microbial community changes caused by its activities. For example, a decrease in salinity is conducive to the colonization of microorganisms and the feeding of animals on litter [49]. We also found that the presence of channeled apple snails significantly reduced the TDS in the water environment. In addition, as a small benthic invertebrate, the abundance of channeled apple snails is higher than that of macrofauna, and they have high population turnover rates. Therefore, their role in the nutrient cycle of sediment cannot be ignored. However, we found that the Chinese mitten crab increased its predation when the proportion of channeled apple snails was higher. Not completely consistent with our prediction, the results of the litter decomposition experiment clearly reflected that the influence of channeled apple snails (whether on water quality, directional change in the microbial community, or decomposition ability) was significantly inhibited by crabs. This suggests that when new species invade, local large predators are very important to maintain the stability of the ecosystem (especially the nutrient cycle) in the invaded area by controlling the density of the invasive species through predation.

There are many studies on the impact of invertebrates on litter decomposition, and most of these experiments have also found that the presence of animals accelerates the process of litter decomposition [50]. However, the interaction between species of natural ecosystems is complex. As we considered the integrity of the brown food chain [51], crabs indirectly slowed the process of nutrient cycling through density-mediated interactions (reducing decomposers). Furthermore, this might also affect the contribution of snails to the green food chain. For example, snails also play a role in the green food chain in the natural freshwater ecosystem, which is not reflected in our simulation experiments. Similarly, Zhang et al. [52] believed that omnivorous crayfish affected ecosystem functions in streams through trophic processing and ecosystem engineering effects (changing debris decomposition, nutrient storage, and shaping benthic communities). Green et al. [53] found that in monopolizing litter processing, red crabs (*Gecarcoidea natalis*) transfer organic matter and nutrients to the soil, thus reducing the available habitat of other animals. Therefore, these large arthropod predators become ecosystem engineers.

In our study, we applied soil zymography, which was previously used to detect soil enzymes in terrestrial ecosystems [54], to the drained substrate of freshwater ecosystems. Compared with traditional enzyme activity detection technology, the largest feature of soil zymography is that it reflects the dynamic characteristics of ecological enzymes from a two-dimensional plane [55]. Our results showed that crabs had stronger bioturbation effects on sediment (Figure 3), which might be due to the more intense movement, predation, and other behaviors of large arthropods. This serious disturbance brought a more obvious balance to the sediment. For example, the existence of crabs made the hotspot area of ecological enzymes in the sediment larger. Biological hotspots represent various sites with higher activity in the ecosystem. For example, the increase in enzyme hotspots reflects the increase in the metabolic rate of microbial reproduction and growth [56]. The results of soil zymography fully showed the top-down effect of benthic arthropods through indirect effects, which could not be provided by traditional research methods. In addition, the grey value image data of the soil zymography showed a significant positive correlation between mass loss of leaf litter and AP activity and hotspot areas. And the vector angle of the enzyme stoichiometry suggests that the sediment primarily has phosphorus limitation. Similarly, experiments on nutrient cycling-related enzymes in forest soils have suggested that we need to pay more attention to changes in phosphorus content and phosphorus cycling-related enzymes [30].

## 5. Conclusions

The diversity of environmental factors and food web structure in ecosystems provides conditions for complex species interactions. Our research showed that a relatively small number of top predators brought direct effects, density-mediated indirect interactions, and trait-mediated indirect interactions to the lower biological community structure and ecological processes. Different interactions may strengthen or offset each other. Overall, these findings imply that macrobenthic arthropods increase the complexity of detrital food webs by regulating biological interactions and enhancing the stability of microbial community structure, and have an important impact on nutrient cycling and energy transfer in freshwater ecosystems. We could use this top-down effect for biological control and engineering construction of freshwater ecosystems.

## Data Availability

All data generated or analyzed during this study are included in this published article and its supplementary information files.

## Supplementary Materials

The following supporting information can be downloaded at: https://www.mdpi.com/article/10.3390/ani13142342/s1. Figure S1: Example of soil zymography. Figure S2: Non-metric multidimensional scaling analysis for taxonomy and carbohydrate-active enzymes. Table S1: Weight of animals in the crab feeding preference experiment. Table S2: Weight of animals in the litter decomposition experiment. Table S3: Water quality parameters of the decomposition system. Table S4: Effects of the treatment, litter type, and mesh size of litter bags on the decomposition rate. Table S5: Mass loss of lotus leaf and standard litter. Table S6: Sediment parameters after 6 weeks of decomposition. Refs. [30,55,57,58,59,60] are cited in Supplementary Materials file.
