# Supplementary material for: Top-Down Effect of Arthropod Predator Chinese Mitten Crab on Freshwater Nutrient Cycling
Source: Animals (Basel). 2023 Jul 18;13(14):2342. doi: 10.3390/ani13142342 (PMC10376719; doi:10.3390/ani13142342)
Supplement: Supplementary file 1 [file animals-13-02342-s001.zip › animals-2459840-supplementary.pdf]

Supplementary Materials

Soil zymography: 4-Methylumbelliferyl phosphate (MUF-P), 4-Methylumbelliferyl β-D-glucopyranoside (MUF-G), and 4-MethyluMbellifery-β-D-cellobioside (MUF-C) were dissolved in a 0.1 mm MES ($C_6H_{13}NO_4SNa_{0.5}$) buffer to prepare a 12 mm substrate solution. We placed the polyamide filter membrane in the substrate solution for 10 min, dried the soaked membrane naturally for 2-5 min, and stuck it tightly to the soil surface. We applied pressure on the membrane to ensure complete contact and incubated it in the dark by shading it with tin foil for 1 h and gently removed the soil particles adhered to it with tweezers. We observed the membrane under a UV lamp (excitation wavelength was 355 nm, emission wavelength was 460 nm). The UV light source, camera, and membrane should maintain a fixed distance and generate images under the same conditions. We used MATLAB R2020a software to analyze the scanned soil zymography images. Each pixel corresponds to a gray value. The substrate concentration in the standard zymography was fitted with the gray value to generate a function. The average enzyme activity and hotspots for soil enzymes were analyzed. The gray image was converted into an RGB color image (Fig. S1) (Cao et al., 2022; Luo et al., 2023).

Hereby, we need to stress that the principle of soil zymography is to detect the fluorescence generated after the reaction of the enzyme and substrate. Enzymes and substrates may diffuse on the polyamide filter membrane. The diffusion is correlated with the water content (Guber et al., 2018). Therefore, special attention should be given to sediment humidity during the detection of enzymes. It cannot be directly immersed in water for testing.

Test of sediment nutrients: We screened the collected sediment samples through a 60-mesh sieve to detect soil organic carbon (SOC) and soil total nitrogen (TN). SOC was determined by the potassium dichromate oxidation-outer heating method, and TN was determined by sulfuric acid catalyst digestion and the Kjeldahl determination method (Ma et al., 2018). These two tests were completed by Nanjing Convinced-test Technology Co., Ltd. In addition, we screened the collected sediment samples through a 100-mesh sieve to detect the soil's total phosphorus (TP). The molybdenum blue method was used to determine the total phosphorus of the soil by a Total Phosphorus Assay Kit (ZC-S0459, Shanghai ZCIBIO Technology Co., Ltd.).

Metagenome sequencing: All sediment samples were stored at −80 °C until DNA extraction. Samples were submitted to Sangon Company (Shanghai, China) for paired-end metagenomic sequencing on an Illumina HiSeq sequencing platform (Zhang et al., 2018; Zheng et al., 2020). The Qubit dsDNA HS assay kit (Q32854, ThermoFisher) was used to accurately quantify genomic concentrations, and then the initial DNA was fragmented using the Covaris ultrasonic DNA crusher (S220, Covaris). Hieff NGS DNA Selection Beads (12601ES56, Shanghai Yisheng Biotechnology Co., Ltd, China) were used to concentrate and retrieve the broken DNA fragments. An NEB Next Ultra DNA Library Prep Kit for Illumina (E7370, NEB) was used to make DNA libraries.

References

Cao, T., Kong, X., He, W., Chen, Y., Fang, Y., Li, Q., Chen, Q., Luo, Y., Tian, X., 2022. Spatiotemporal characteristics of enzymatic hotspots in subtropical forests: In situ evidence from 2D zymography images. Catena 216, 106365.

Guber, A., Kravchenko, A., Razavi, B.S., Uteau, D., Peth, S., Blagodatskaya, E., Kuzyakov, Y., 2018. Quantitative soil zymography: Mechanisms, processes of substrate and enzyme diffusion in

porous media. Soil Biology and Biochemistry 127, 156-167.

Luo, Y., Wang, L., Cao, T., He, W., Lu, S., Li, F., Zhang, Z., Chang, T., Tian, X., 2023. Legacy effect of plant chemical defence substances on litter decomposition. Plant and Soil 487, 93–108.

Ma, T., Zhu, S., Wang, Z., Chen, D., Dai, G., Feng, B., Su, X., Hu, H., Li, K., Han, W., Liang, C., Bai, Y., Feng, X., 2018. Divergent accumulation of microbial necromass and plant lignin components in grassland soils. Nature Communications 9, 3480.

Zhang, Y.S., Jiang, J.Q., Zhao, Q.L., Wang, K., Yu, H., 2018. Analysis of functional genomes from metagenomes: Revealing the accelerated electron transfer in microbial fuel cell with rhamnolipid addition. Bioelectrochemistry 119, 59-67.

Zheng, M.Q., Shi, J.X., Xu, C.Y., Han, Y.X., Zhang, Z.W., Han, H.J., 2020. Insights into electroactive biofilms for enhanced phenolic degradation of coal pyrolysis wastewater (CPW) by magnetic activated coke (MAC): Metagenomic analysis in attached biofilm and suspended sludge. Journal of Hazardous Materials 395.

Supplementary Figure

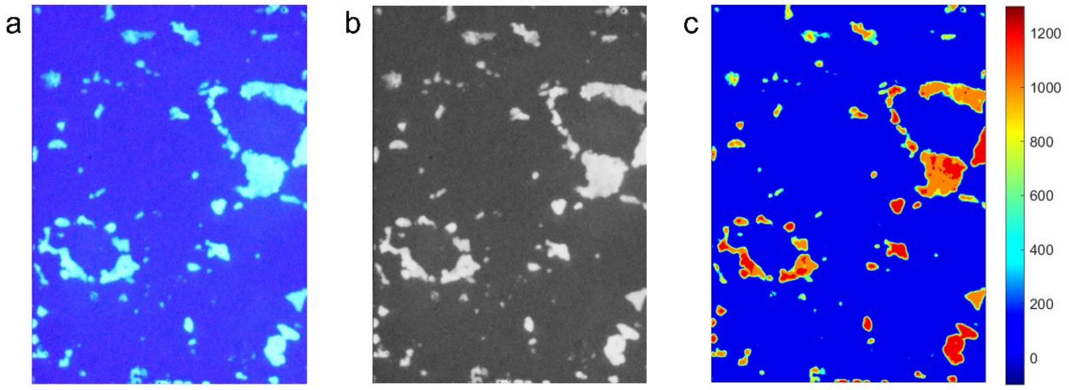

Figure S1. Example of soil zymography.

The data extraction method of soil zymography. (a) The original scanned image. (b) The gray value image. The scanned image is analyzed by MATLAB, and each pixel corresponds to a gray value. (c) An RGB color image. The substrate concentration in the standard enzyme spectrum is fitted with the gray value to generate a function, and the gray image is converted into color. The average enzyme activity and the hotspots 25% higher than the average were calculated by MATLAB. The unit is pmol·cm^{-2}·h^{-1}.

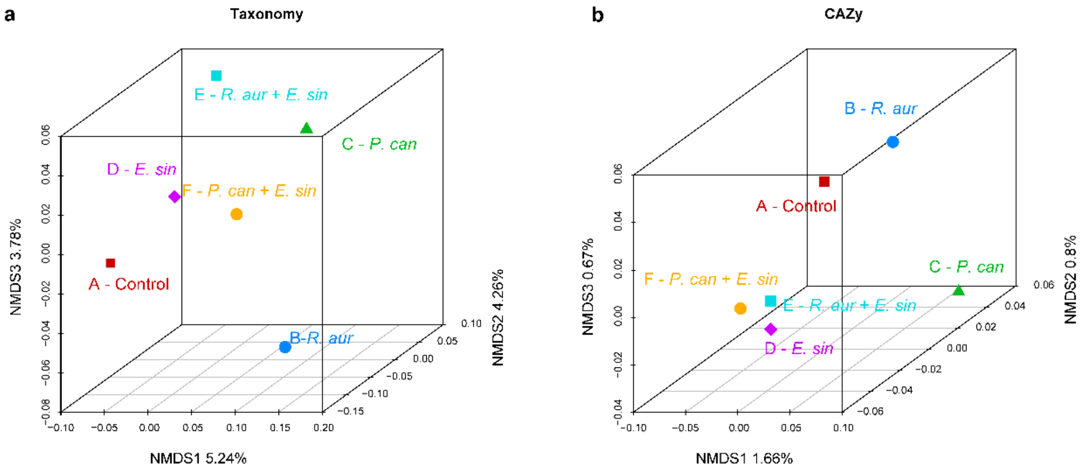

Figure S2. Non-metric multidimensional scaling analysis for taxonomy and carbohydrate-active enzymes. (a) The NMDS result for taxonomy. (b) The NMDS result for CAZy. CAZy is a specialist database of carbohydrate-active enzymes, including enzymes that catalyze the degradation, modification, and biosynthesis of carbohydrates. Sample sites were relatively more consistent and concentrated in the treatments where crabs were involved (Treatment D, E, and F).

Supplementary Table

Table S1. Weight of animals in the crab feeding preference experiment.

Treatment	No.	*Radix auricularia*			*Pomacea canaliculata*			*Eriocheir sinensis*	
		n	Mean (g)	SE	n	Mean (g)	SE	n	Weight (g)
Experimental	1	5	0.6598	0.0447	5	0.6682	0.0719	1	41.86
	2		0.6009	0.0570		0.5974	0.0915		40.99
	3		0.5791	0.0868		0.5733	0.1097		37.10
	4		0.4820	0.0281		0.4901	0.1225		33.63
	5		0.4062	0.0305		0.4102	0.0735		28.41
	6		0.4116	0.0372		0.4099	0.1062		28.26
	7		0.5619	0.0225		0.5567	0.1082		37.81
	8		0.4512	0.0550		0.4652	0.0655		36.49
	9		0.4617	0.0607		0.4751	0.0549		35.38
	10		0.4700	0.0299		0.4822	0.0590		33.11
	11		0.5486	0.0510		0.5425	0.0856		45.05
	12		0.5460	0.0446		0.5514	0.0839		37.11
	13		0.4442	0.0076		0.4585	0.0895		28.86
	14		0.6166	0.0337		0.6071	0.0354		24.49
	15		0.4429	0.0380		0.4542	0.0661		29.08
	16		0.3246	0.0160		0.3171	0.0271		34.44
	17		0.4486	0.0430		0.4369	0.0505		40.43
	18		0.3698	0.0359		0.3735	0.0899		34.81
	19		0.3313	0.0103		0.3375	0.0234		28.87
	20		0.4635	0.0644		0.4658	0.0919		34.02
Control	21	5	0.3502	0.0217	5	0.3574	0.0440	0	
	22		0.3453	0.0251		0.3606	0.0408		
	23		0.3725	0.0182		0.3695	0.0698		
	24		0.3487	0.0273		0.3507	0.0722		
	25		0.3718	0.0332		0.3654	0.0783		
	26		0.3017	0.0191		0.3046	0.0313		
	27		0.3406	0.0509		0.3296	0.0138		
	28		0.3213	0.0130		0.3077	0.0694		
	29		0.2797	0.0172		0.2460	0.0235		
	30		0.2849	0.0199		0.2831	0.0075		
	31		0.2850	0.0324		0.3025	0.0271		
	32		0.2969	0.0157		0.3109	0.0411		
	33		0.3432	0.0478		0.3385	0.0723		
	34		0.4128	0.0513		0.4043	0.0643		
	35		0.3847	0.0182		0.3808	0.0890		
	36		0.4067	0.0382		0.3990	0.0708		
	37		0.3221	0.0179		0.3092	0.0588		
	38		0.3201	0.0155		0.3001	0.0682		
	39		0.3231	0.0263		0.3550	0.0896		
	40		0.3559	0.0147		0.3298	0.0775		

The average weight of *R. auricularia* in each water tank ≈ that of *P. canaliculata*. The Mann–Whitney U test was performed for each group of data ($P > 0.05$).

Table S2. Weight of animals in the litter decomposition experiment.

Species	Treatment	n	Mean (g)	SE
Eriocheir sinensis	D	5	38.0520	1.9989
	E		38.1060	1.3747
	F		38.0900	1.0361
Radix auricularia	B	180	0.8574	0.0315
	E		0.8582	0.0271
Pomacea canaliculata	C	180	0.8564	0.0399
	F		0.8549	0.0428

The average weight of *R. auricularia* ≈ that of *P. canaliculata*. The Mann–Whitney U test was performed for each group of data ($P > 0.05$).

Table S3. Water quality parameters of the decomposition system.

Water index	Group											
	A		B		C		D		E		F	
pH	8.15±0.02	a	7.86±0.02	d	7.85±0.01	d	7.93±0.02	c	7.95±0.01	c	8.03±0.01	b
COND ($ms \cdot m^{-1}$)	0.82±0.01	c	0.83±0.01	c	0.76±0.00	d	0.84±0.01	bc	0.86±0.01	b	0.93±0.01	a
TDS ($mg \cdot L^{-1}$)	576.11±6.48	d	580.50±5.60	cd	529.57±3.28	e	590.43±5.59	bc	603.70±3.63	b	652.86±4.27	a
SIR (%)	0.14±0.02	b	0.23±0.03	a	0.22±0.02	a	0.15±0.01	b	0.16±0.01	b	0.19±0.02	ab

The value is the mean ± SE. The number of replicates of pH, COND, and TDS in each treatment is n = 44 (tested once a day), and that of SIR is n = 6 (tested once a week). Lowercase letters are LSD test results, and different letters in the same water quality parameter (the same line) represent significant differences.

Table S4. Effects of the treatment, litter type, and mesh size of litter bags on the decomposition rate.

		Estimate	SE	t value	P	
Intercept		0.326	0.022	15.147	**<0.001**	***
Group	A	0	-			
	B	0.117	0.025	4.696	**<0.001**	***
	C	0.170	0.025	6.832	**<0.001**	***
	D	0.066	0.025	2.656	**0.009**	**
	E	0.041	0.025	1.645	0.102	
	F	0.038	0.025	1.515	0.132	
Litter	Cotton	0				
	Leaf	-0.025	0.018	-1.403	0.162	
	Wood	-0.324	0.018	-18.467	**<0.001**	***
Bag	Large	0				
	Small	-0.061	0.014	-4.221	**<0.001**	***

Significant relationships are indicated by bold P values.
* P<0.05   ** P<0.01   *** P<0.001

Table S5. Mass loss of lotus leaf and standard litter.

Litter		Group											
Material	Bag aperture	A		B		C		D		E		F	
Leaf	0.2 mm	30.21±1.48	bc	30.57±0.73	abc	31.31±2.16	abc	27.21±0.83	c	34.05±2.00	ab	35.48±1.53	a
	8 mm	34.47±2.21	b	40.46±5.30	b	82.81±6.71	a	85.43±3.54	a	39.18±1.54	b	34.19±1.14	b
Sliver	0.2 mm	25.94±1.13	d	69.81±2.97	a	55.79±0.99	b	32.52±1.65	cd	33.89±1.46	c	34.12±2.98	c
	8 mm	26.01±1.70	d	75.45±3.53	b	98.51±1.33	a	27.46±1.04	d	34.99±1.00	c	37.61±1.61	c
Wood	0.2 mm	2.68±0.11	b	4.76±0.72	a	5.01±0.27	a	4.39±0.23	a	5.53±0.40	a	4.86±0.47	a
	8 mm	2.85±0.67	b	3.60±0.79	ab	4.44±0.30	ab	4.79±0.46	a	5.33±0.40	a	4.58±0.62	ab

The value is the mean ± SE. The unit in percentage (%). Lowercase letters are LSD test results, and different letters in the same decomposition system (the same line) represent significant differences.

Table S6. Sediment parameters after 6 weeks of decomposition.

Soil index	Group											
	A		B		C		D		E		F	
COND ($ds\cdot m^{-1}$)	0.06±0.01	ab	0.08±0.01	a	0.04±0.01	b	0.06±0.01	ab	0.05±0.01	ab	0.70±0.02	ab
SIR (%)	0.21±0.00	b	0.23±0.01	ab	0.22±0.01	ab	0.22±0.01	ab	0.25±0.01	a	0.22±0.01	b
pH	8.75±0.02	a	8.66±0.01	c	8.68±0.03	bc	8.76±0.02	a	8.72±0.00	ab	8.68±0.02	bc
AP (grey value)	93.91±1.22	b	93.52±0.73	b	94.32±1.09	b	129.80±10.77	a	138.08±13.70	a	130.36±11.47	a
AP.hotspot (%)	5.80±0.75	b	4.93±0.23	b	3.16±0.49	b	19.94±4.62	a	22.00±5.49	a	21.80±4.89	a
BG (grey value)	82.87±0.47	c	83.64±0.92	c	83.90±0.74	c	116.13±8.29	b	118.10±13.08	b	184.32±7.19	a
BG.hotspot (%)	4.21±0.36	bc	2.98±0.48	c	3.28±0.62	bc	19.72±4.38	a	21.66±6.65	a	18.85±8.43	ab
NAG (grey value)	79.98±1.40	a	79.37±0.73	a	80.06±0.32	a	94.74±5.67	a	83.81±2.50	a	98.14±13.57	a
NAG.hotspot (%)	3.76±0.77	bc	3.06±0.94	bc	1.91±0.47	c	11.98±3.17	ab	6.52±1.56	abc	14.50±6.10	a
lnBG/lnNAG	1.01±0.00	c	1.01±0.00	c	1.01±0.00	c	1.04±0.02	bc	1.07±0.02	b	1.15±0.03	a
lnBG/lnAP	0.97±0.00	b	0.98±0.00	b	0.97±0.00	b	0.98±0.01	b	0.97±0.02	b	1.08±0.02	a
lnNAG/lnAP	0.96±0.01	a	0.96±0.00	a	0.96±0.00	a	0.94±0.02	ab	0.90±0.02	b	0.94±0.02	ab
Vector length	1.40±0.00	b	1.41±0.00	b	1.40±0.00	b	1.43±0.01	b	1.44±0.03	b	1.58±0.03	a
Vector angle (°)	46.03±0.18	b	46.05±0.04	b	46.05±0.09	b	46.86±0.70	ab	47.90±0.66	a	46.88±0.53	ab
SOC ($g\cdot kg^{-1}$)	9.62±0.18	ab	10.12±0.34	a	9.30±0.22	b	9.54±0.35	ab	9.86±0.08	ab	9.24±0.10	b
TN ($g\cdot kg^{-1}$)	0.96±0.02	a	0.95±0.02	a	0.95±0.03	a	0.91±0.01	ab	0.92±0.02	a	0.86±0.01	b
TP ($g\cdot kg^{-1}$)	2.06±0.19	a	1.25±0.34	a	1.12±0.25	a	1.95±0.19	a	1.86±0.33	a	2.04±0.44	a
C/N	10.03±0.21	ab	10.67±0.21	ab	9.83±0.24	b	10.48±0.36	ab	10.75±0.27	a	10.81±0.22	a
C/P	4.90±0.21	b	15.38±6.81	a	10.46±2.12	ab	5.06±0.37	b	6.57±1.51	ab	6.51±1.92	ab
N/P	0.49±0.05	a	1.41±0.59	a	1.09±0.25	a	0.49±0.05	a	0.63±0.16	a	0.62±0.19	a

The value is the mean ± SE, and the number of replicates of each treatment is n = 5. Lowercase letters are LSD test results, and different letters in the same sediment parameter (the same line) represent significant differences.
